# Solidification/Stabilization Technology for Radioactive Wastes Using Cement: An Appraisal

**DOI:** 10.3390/ma16030954

**Published:** 2023-01-19

**Authors:** Ismail Luhar, Salmabanu Luhar, Mohd Mustafa Al Bakri Abdullah, Andrei Victor Sandu, Petrica Vizureanu, Rafiza Abdul Razak, Dumitru Doru Burduhos-Nergis, Thanongsak Imjai

**Affiliations:** 1 Department of Civil Engineering, Shri Jagdishprasad Jhabarmal Tibrewala University, Rajasthan 333001, India; 2 Center of Excellence Geopolymer and Green Technology (CEGeoGTech), Universiti Malaysia Perlis (UniMAP), Perlis 01000, Malaysia; 3 Faculty of Chemical Engineering Technology, Universiti Malaysia Perlis (UniMAP), Perlis 01000, Malaysia; 4 Faculty of Material Science and Engineering, Gheorghe Asachi Technical University of Iasi, 41 D. Mangeron St., 700050 Iasi, Romania; 5 Romanian Inventors Forum, Str. Sf. P. Movila 3, 700089 Iasi, Romania; 6 National Institute for Research and Development for Environmental Protection INCDPM, 294 Splaiul Independentei, 060031 Bucharest, Romania; 7 Technical Sciences Academy of Romania, Dacia Blvd 26, 030167 Bucharest, Romania; 8 School of Engineering and Technology, Walailak University, Nakhon Si Thammarat 80160, Thailand

**Keywords:** radioactive wastes, cement, solidification/stabilization (S/S), biochar, waste form, supplementary cementitious materials (SCM), magnesia-based cement, calcium sulphoaluminate cement, calcium aluminate cement

## Abstract

Across the world, any activity associated with the nuclear fuel cycle such as nuclear facility operation and decommissioning that produces radioactive materials generates ultramodern civilian radioactive waste, which is quite hazardous to human health and the ecosystem. Therefore, the development of effectual and commanding management is the need of the hour to make certain the sustainability of the nuclear industries. During the management process of waste, its immobilization is one of the key activities conducted with a view to producing a durable waste form which can perform with sustainability for longer time frames. The cementation of radioactive waste is a widespread move towards its encapsulation, solidification, and finally disposal. Conventionally, Portland cement (PC) is expansively employed as an encapsulant material for storage, transportation and, more significantly, as a radiation safeguard to vigorous several radioactive waste streams. Cement solidification/stabilization (S/S) is the most widely employed treatment technique for radioactive wastes due to its superb structural strength and shielding effects. On the other hand, the eye-catching pros of cement such as the higher mechanical strength of the resulting solidified waste form, trouble-free operation and cost-effectiveness have attracted researchers to employ it most commonly for the immobilization of radionuclides. In the interest to boost the solidified waste performances, such as their mechanical properties, durability, and reduction in the leaching of radionuclides, vast attempts have been made in the past to enhance the cementation technology. Additionally, special types of cement were developed based on Portland cement to solidify these perilous radioactive wastes. The present paper reviews not only the solidification/stabilization technology of radioactive wastes using cement but also addresses the challenges that stand in the path of the design of durable cementitious waste forms for these problematical functioning wastes. In addition, the manuscript presents a review of modern cement technologies for the S/S of radioactive waste, taking into consideration the engineering attributes and chemistry of pure cement, cement incorporated with SCM, calcium sulpho–aluminate-based cement, magnesium-based cement, along with their applications in the S/S of hazardous radioactive wastes.

## 1. Introduction

Arthur Schopenhauer, a great German philosopher, once stated that “each and every single truth passes all the way through three phases prior to being documented, at first, it is ridiculed; secondly, it is aggressively opposed, and in third and ultimate stage, it is recognized as being self-evident”. For the last more than three decades, this is exactly a fitting statement in the context of the history of stabilization/solidification (S/S) technology. The S/S of perilous and death-defying radioactive wastes provides a grand necessitate in the field of civil engineering with a view to consolidating the research, and general practices in high-tech work and especially in technology. Simply speaking, solidification/stabilization (S/S) technology can lend a hand to make the radioactive wastes physically more stable in order to manage them under atmospheric conditions.

### 1.1. Radioactive Wastes

Nuclear energy, i.e., atomic energy, is that energy which lies in the nucleus of an atom, which is not only a promising non-fossil-based energy source but also exhibits minimal emissions of carbon dioxide (CO_2_) [1,2] proving it more beneficial [3,4]. In developing nations such as China, there is a foreseeable development of nuclear energy in the coming decades owing to the exigency for energy boosts and a net zero carbon footprint policy [3,4]. Therefore, nuclear energy is heading towards being established as one of the alternatives that may lend a hand to mankind to address the global energy crisis in the upcoming time. In 2018, the production of nuclear power was reported as more or less 10.2% of the total electricity generation in the world and it is likely to reach 13% in 2030 [5]. Radioactive wastes are generated from several sources, viz., nuclear power plants, nuclear armament, or nuclear fuel treatment plants, however, the majority of waste originates from the nuclear weapons reprocessing and nuclear fuel cycle. The nuclear industries are generating radioactive wastes during different processes of production and that is the reason for the accumulation of roughly 200,000 m^3^ of nuclear waste with low- and intermediate-level global generation each year, in harmony with the statistics of the World Nuclear Energy Association [6]. Consequently, the development of safer and competent low- as well as intermediate-level radioactive waste treatment technology has turned out to be a great dilemma for nuclear power plants. The low-level radioactive wastes, i.e., the technical wastes, generated during the processes of maintenance include incompressible and compressible components, viz., plastic, absorbent paper, gloves, rags, scrap work clothes and gas jackets, which account for about 90% of the total volume of radioactive nuclear waste. The bulk quantity of the low-level radioactive wastes in nuclear power plants can be classified as combustible wastes; as a result, the thermal treatment technologies could lend a hand to accomplish higher volume reduction, inorganic alteration and lower residue radioactivity levels. Amongst the thermal treatment technologies, counting incineration, melting, solidification and molten salt-oxidation technology, dry oxidation as well as thermal plasma [7], dry oxidation technologies such as pyrolysis and gasification could attain a higher volume reduction of low-level radioactive wastes with the safer treatment of radionuclide [8].

Several nuclear reactors with graphite as a moderator and reflector are facing being decommissioned sooner or later, and the waste of radioactive graphite is a huge part of the concerned wastes. By 2021, more than 2.5 million tons of irradiated graphite, i.e., i-graphite, generated from nuclear reactors, is for the time being stored in provisional storage facilities and reactor stores [9]. According to International Atomic Energy Agency (IAEA) [10], the i-graphite inventory is chiefly concerted in USA, Russia and UK. The process of production of nuclear power generates radioactive wastes of low-level radioactive waste (LLW), intermediate-level radioactive waste (ILW) and high-level radioactive waste (HLW) depending on its radioactivity attributes. The radionuclide present in the radioactive wastes is for the most part made up of 235U and its fission yields, namely, 137Cs, 90Sr, 60Co,140Ba, 129I, etc. Amongst them, the half-life periods of 137Cs and 90Sr are the greatest figures at 33 and 29.9 years, in that order. The radiation occurring all throughout their decay will gravely contaminate the ecosystem and threaten the life safety measures of lives on the planet. The conventional management move towards radioactive wastes is to solidify them first of all and after that dispose of them below the subsurface or in deep geological dumping sites. The quantity of the low and intermediate radioactive waste liquids is much bigger in comparison with the high radioactive waste liquid, which accounts for beyond 90% of the total radioactive wastes. The technique for cement solidification is far and wide employed in the solidification treatment of low-level and intermediate-level radioactive liquids because of the saving of raw material and its uncomplicated progression [11,12]. Two huge Chinese nuclear plants are exercising cement solidification technology to cope up with low- and intermediate-level radioactive waste liquids for lots of years successfully and lucratively. During decommissioning of nuclear power plants and their operation, the generation of low- and intermediate-level radioactive wastes takes place. A large quantity of this waste is dumped—the nonconforming part that necessitates solidification and packaging, which is hard to decontaminate and mainly encloses dispersive particulate wastes such as the debris of spent concretes, slurry or sludge, and fine-grained polluted soil. The published report of IAEA has focused on such dispersive/particulate radioactive wastes and is directed to treat and package them correctly for permanent clearance [13]. Quite a lot of stabilization/solidification techniques were developed to treat the referred radioactive wastes with cement, polymer and asphalt. The said agents of solidification alter the dispersive/particulate wastes into non-dispersive solidified wastes, formed all the way through their definite process [14]. In the meantime, the key setback is a noteworthy boost in their volume when the solidification of the dry particles (powder) is performed by grouping with cement, asphalt, or polymer because each particulate matter becomes encapsulated and goes through the solidification agent [15]. This augment in the volume increases the cost of waste disposal and the capability of disposal facilities is also filled up swiftly. With a view to addressing the referred issues, the investigations on minimizing the volume of the powdered wastes and the solidification technology are essential.

Primarily, Liquid Radioactive Waste (LRW) is found generated in the nuclear power industry and also in unexpected nuclear accidents. Its immobilization is one of the most efficient measures for managing radioactive waste. In order to generate liquid radioactive waste, nuclear waste is dissolved in boiling nitric acid (HNO_3_), and both uranium (U) and plutonium (PUREX) are recovered. The leftover liquid is considered liquid radioactive waste, which contains radioactive substances, namely, Cs^+1^, Sr^+2^, and Co^+2^. Customarily, the liquid radioactive waste is managed in three stages [16,17]:

- Cooling the leftover liquid;
- Drying and concentrating;
- Mixing it with silicate or borate.

**Figure 1:**
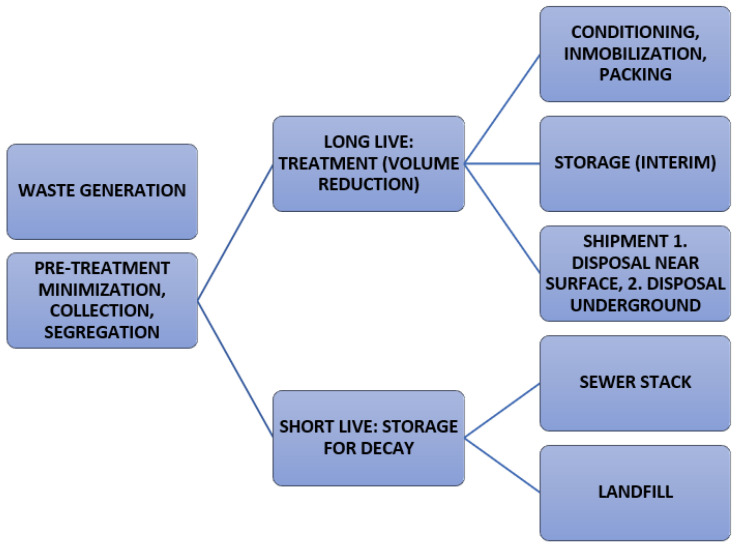
Radioactive waste management chart.

The waste materials are acquired in a form of glass at towering temperatures. In the context of 90Sr in liquid radioactive waste, there are some moves to separate it from LRW, including ion exchange, precipitation and solvent extraction [18]. Alternatively, a solid matrix can immobilize 90Sr. Every single technique should spotlight the application of low-priced energy. Globally, the liquid radioactive waste immobilization in solid products has been well-studied [19,20,21]. The chemical immobilization of Sr^+2^ in calcium–silicate–hydrate (C–S–H) does not occur, and this is the disadvantageous root cause of the application of traditional Portland cement to immobilize Sr^+2^ ions in this medium [22]. The phosphate has an ideal effect in the solidification of Sr^+2^, assuggested in preceding investigations. For this reason, phosphate-based materials are regarded as ideal matrices for the aqueous immobilization of Sr^+2^. The radioactive waste management chart is depicted in Figure 1.

### 1.2. Stabilization/Solidification (S/S) Technology

“Stabilization” refers to the chemical methods, which can mitigate the perilous potential of a particular waste by converting the pollutants into less soluble, poisonous or movable forms. However, the stabilization does not essentially alter the handling attributes and physical nature of the definite type of waste. On the other hand, “Solidification” refers to the methods of encapsulation of the wastes, which form a solid material. However, the solidification does not essentially engage a chemical interaction amongst the pollutants and the hardening additives. The S/S can be accomplished through chemical reaction kinetics among hardening reagents and the waste, or by means of courses of action of a mechanical kind. The waste form or the solidification produced might be a clayey or argillaceous material, a granular particulate, or in the form of a monolithic block, as well as a few other physical forms, which are normally regarded as “solid.” The migration of pollutants is frequently confined via coating the wastes with the help of materials having inferior permeability, or by slimming down the surface area exposed to leaching. Frequently, both terms are referred to as S/S and can be utilized interchangeably. Inorganic binders such as cement are brilliantly efficient for immobilizing heavy metals using mechanisms of physical and chemical containment. Significantly, loads of substances present in the wastes influence the setting and solidifying attributes of the binders, in particular, the cement-based systems for cementing. There are varied processes and equipment, which are developed to serve the purposes. The ex situ or in situ processes can be useful to carry out the grouping of binders and wastes, of which, the in situ techniques are getting much positive response for the remediation of polluted sites, and they can be further categorized as backhoe-based methods, shallow area methods and drilling, augering, jetting, or trenching methods. While, in the case of ex situ processes, the mixing, mortar mixers, pug mills, or concrete mixers are mostly utilized. The depth of the pollution and the attributes of the tainted media are regarded as the bases for the choice of the kind of mixing method.

As we know, the inappropriate management of unsafe radioactive wastes creates a solemn threat to human health and other breathing organisms and their surroundings. For illustration, the toxic leachates, which include perilous wastes from unacceptably maintained unsafe waste landfill, could rigorously pollute the subsurface and surface waters; leakage or accidents of nuclear power plant blasts can gravely kill or injure the neighboring public. Therefore, these waste management projects must be regulated with adequately knowledgeable, competent, and reliably dependable regulations and legislation. The fundamentals of the management of risky, radioactive, and mixed wastes are essential for enough definition, classification, designation, and characterization to offer bounds to the crisis, which vary from nation to nation. Since 1980, the U.S. Environmental Protection Agency (EPA) has developed an all-inclusive program for perilous waste to make certain that risky waste can be managed unharmed. A “cradle-to-grave” strategy for such wastes from the point of generation to final removal is established for their identification, recycling, storage, and dumping. In 1976, RCRA, i.e., The Resource Conservation and Recovery Act, was motivated by apprehension over the indecent discarding of hazardous wastes. RCRA Subtitle C provides a wide-ranging program concerning the identification, generation, transportation, treatment, storage, and safe removal of risky wastes. The Atomic Energy Act (AEA) of 1954 is the fundamental law governing the production, utilization, possession, accountability, and disposal of radioactive materials in the U.S.A. Additionally, several laws state radioactive waste management methods and authorities such as the Low-Level Radioactive Waste Policy Act (LLWPA) and the Nuclear Waste Policy Act (NWPA). The Nuclear Regulatory Commission (NRC) or the U.S. Department of Energy (DOE) under the AEA regulates hazardous radioactive wastes. The characterization and classification of radioactive wastes are done by and large specified by related regulations and laws.

Characteristically, stabilization/solidification (S/S) is a process that entails the mixing of the waste with a binder in order to slim down the contaminant leachability by both chemical and physical means and to transform the perilous waste into an eco-acceptably waste form for landfill dumping or construction utilizations. The S/S is extensively employed to dispose of low-level radioactive wastes (LLRW), hazardous, and mixed wastes, as well as remediation of tainted sites. The S/S is regarded as the Best Demonstrated Available Technology (BDAT) for 57 harmful wastes in accordance with The United States Environmental Protection Agency (USEPA) [23]. The report of the USEPA for 1996 revealed that more or less 30% of the Superfund remediation sites applied S/S technologies [24]. Cementitious materials are the most commonly exercised for S/S among all the binders. The cement solidification method is extensively employed in the solidification treatment of the LLW and ILW liquids due to the economy of raw material and its easy process [11,12]. Two large nuclear plants utilizing cement solidification technology are located in China, to deal with lower and medium radioactive waste liquids for several years and, they are proven to be flourishing and money-spinning. Considering the many benefits, hydraulic cement is extensively employed for S/S of low-level radioactive wastes, perilous wastes, mixed wastes, and in the remediation of polluted sites. The cement-based S/S technology exhibits the following benefits in comparison with the rest of the other technologies [25]: (1) excellent impact and compressive strength, (2) good-quality and long-term physical and chemical stability, (3) relatively cost-effective, (4) documented application and compatibility with various wastes over decades, (5) familiar material and technology, (6) extensive accessibility of the chemical ingredients, (6) non-toxicity of the chemical ingredients, (7) effortlessness of application in processing because usually carried out at ambient temperature and pressure and no need of unique or very special equipment at all, (8) higher loadings of waste are feasible, (9) inert to ultraviolet (UV) radiation, (10) elevated resistance to bio-degradation, (11) lower water solubility and leachability of some pollutants, (12) comparatively lower water permeability, (13) capacity of most aqueous wastes to bind chemically with matrix, (14) good mechanical and structural attributes, (15) good self-guarding for radioactive wastes, (15) fast, controllable setting, with no segregation or settling during curing, (16) absence of free water provided correctly formulated, and (17) a longer shelf-life of cement powder.

The S/S of pollutants using cements includes the following three features:

(1) The chemical fixation of pollutants—chemical interactions among the cement hydration yields and the pollutants;
(2) The physical adsorption of the pollutants on the surface of cement hydration yields;
(3) The physical encapsulation of polluted waste or soil.

Of these, Items1 and 2 rely upon the nature of the yields of hydration and pollutants while Item 3 relates to both the nature of the hydration yields as well as the paste density and its physical structure. The cement-based waste forms may be fitting for controlled construction exercises provided the leaching and other performance of the cement-solidified waste forms meet suitable eco-criteria.

For the S/S of waste, the choice of cementing materials must be made considering the following criteria on the basis of the waste properties:

(1) The cement and the waste compatibility;
(2) The pollutant’s chemical fixation;
(3) The physical encapsulation of polluted waste and soil;
(4) The durability of ultimate waste forms;
(5) The waste form leachability;
(6) The gainfulness of S/S in terms of cost.

Practically, numerous additives are frequently employed with cementing materials to resolve all of the above aspects.

Quite recently, the application of cementitious materials for the solidification of dangerous matters has proven to be significantly promising. The benefits of solidification/stabilization include the following [26]:

- Safe transport and easy burial;
- Enhanced physical attributes of the wastes for effortless handling;
- Lesser eco-pollution by leaching and evaporation of risky constituents;
- Potential for recycling wastes into construction material;
- Detoxification of substances for safe-guarding workers.

The Portland cement, correct additives and in some incidents fly ash are grouped together with the waste with a view to produce a solidified mass for dumping during solidification. A gel is initially developed, then fibrils formed as silicate compounds hydrate when the waste is grouped together with cement. The referred inter-locking fibrils bind various hydration yields and the cement into a hardened mass. The process of solidification using the Portland cement is most appropriate to inorganic wastes such as incinerator residue, heavy metal enclosing wastes and road wastes. The keeping of metals in the form of insoluble hydroxide of carbonate salts is assigned to the higher pH of the cement. Whereas plastic, metal filings, and asbestos-like materials boost the strength of the matrix, other organic and inorganic compounds can retard setting, decline the final strength and cause swelling. The additives enclosing clay, sodium silicate and vermiculite may be integrated with the mixes to counterbalance the influences of the said materials. Additionally, the low-level radioactive wastes and organic wastes can be solidified using Portland cement.

The S/S is a potentially promising technology that utlizes the supplement of a binding agent to encapsulate and trim down the mobility of the dangerous waste elements [27]. The S/S can act as a significant potential process for making wastes acceptable for land dumping, since the constraints on filling the lands turn out to be stronger and wastes particularly hazardous ones are banned from land dumping. Inferior permeability and lesser pollutant leaching rates can make the forbidden wastes acceptable for their disposal in landfills subsequent to the S/S process [28]. In the S/S process, the method of using Portland cement as a binding agent mixed with water and the heat has been developed. The mixture turns out to be strongly alkaline. The chemical reactions become sluggish after a few minutes. After that, an induction period or dormant period usually lasts for several hours. The anhydrous clinker grains develop as coating proceeds, with an early amorphous precipitate throughout the initial reaction period, which plays a role of semi-protective film and slows reaction during the induction period. The breakdown of the film marks the onset of swift hydration towards the finish of the induction period. Also, the breakdown of the film initiates the growth of a constant but initially lower strength gel network of linking particles, ensuing in physical hardening of the cement matrix. As the gel carries on the process of solidification and densification, the cement achieves maximal strength. The archetypal contemporary Portland cement attains around two thirds of hydration in 28 days [29]. The chief chemical that is regarded in hydrated cement is colloidal calcium–silicate–hydrate (C-S-H) gel. This gel is developed at the surfaces of particles of cement [30]. The C-S-H gel has significant implications for the mechanisms of fixation during the process of solidification and it is largely accountable for strength development [31,32].

Most frequently, HE employs cement as a binder for an assortment of wastes for solidification. The utilization of cement for solidification is beneficial for the simplicity of the process and it taking place under normal temperature, however, the quite high cost of this binder and also the eco-aspect of anthropogenic CO_2_ footprints associated with cement production are the prominent setbacks [33,34,35]. The said process is apposite for inorganic materials, viz., ashes and dehydrated sludge from industrial wastewater treatment plants, and also for the solidification of waste going to landfills. At present a broad range of combinations of diverse kinds of binders is being exercised. The cement itself is an energy-intensive product; as a result, it is essential to ensure the optimum composition of the solidification mix. The drawback lies with the sensitivity to the presence of definite substances that influence the hydration reaction kinetics and the solid structure development. There are other disadvantages, such as a boost in the volume of solidified waste, which is unsuitable for depositing in landfills, as well as a low resistance to corrosion agents. More often than not, the wastes are mixed with Portland cement and additives, which affect, in a positive way, the characteristics of the cement, and with enough water content, this begins the hydration reaction kinetics. Subsequently, the S/S process initiates and waste is added into the cement structure. The waste reacts with water and cement to develop hydroxides of metals or carbonates. Normally, the same are less soluble than the original metal compounds in the waste. The cementation technology can predominantly be executed on the accessible equipment—solidification technology lines (mobile/stable). The cement can be employed as an activator for other potentially binding materials, e.g., low-priced fly ashes or glassy slags. Ultimately, the referred to secondary binders have turned out to be an integral part of the cement matrix, which uses one kind of waste to immobilize other sorts of more hazardous wastes. The development of less soluble hydroxides of metals or carbonates all through the hydration reactions results in meeting the requisite limits for leachability examinations. The benefit of solidification technology also lies in the likelihood of processing amorphous metals. Also, solidified/stabilized waste can be managed with no trouble and the danger of dust creation is very little. The discharge of heavy metals from the product is also moderately lower. The output solidification product can often be exercised as construction material or backfill in transport construction engineering or mining operations [36].

Naturally, radioactive elements are found in the crust of the earth. The large unstable atoms turn out to be more stable ones by emitting radiation to eliminate surplus atomic energy, i.e., radioactivity. The said radiation can be emitted in the form of positively charged (+ve) alpha and negatively charged (−ve) beta particles, as well as gamma or X-rays. The radiation from radioactive materials of alpha and gamma rays affect the body very badly. The waste materials that either enclose or get contaminated with radionuclide at concentrations or activities beyond nationwide regulatory authority-established clearance levels for which no application is at this time predicted are included under the roof of “radioactive wastes”. In other words, radioactive waste comprises of any material which is either inherently radioactive, or gets contaminated with a dose of radioactivity, and that is deemed to have no more utilization. Characteristically, an amount of radioactive waste consists of numerous radionuclides that are unstable isotopes of elements, which experience decay and thereby emit ionizing radiation. The said radiation is very much injurious to humans and the eco-system. The diverse isotopes emit dissimilar kinds and levels of radiation that persist for unlike periods of time. However, the radioactive nature of all radioactive waste grows weaker with time. Every radionuclide present in the waste possesses a half-life, i.e., the time it uses for half of the atoms to decay into another nuclide. Ultimately, all radioactive waste decays into non-radioactive elements, meaning “stable nuclides”. Thus, the radioactive wastes decay naturally over the time similar to all radioactive material. Therefore, once the radioactive material decays adequately, the waste is regarded as non-hazardous; however, the time ranges widely for the purpose right from a few hours to thousands of years, as found in the case of plutonium (Pu), which is highly radioactive. These wastes are being produced by industries, namely, in the fields of nuclear power generation, mining, mineral exploration, medicine, agriculture, manufacturing, defense, definite kinds of scientific researches, non-destructive testing, and reprocessing of nuclear weapons. It is a known fact that the nuclear power is pigeonholed by the very huge quantity of energy extended from a very little quantity of fuel, and the amount of waste generated throughout this progression is also comparatively tiny. Nevertheless, the bulk of the waste generated is radioactive and consequently it must be cautiously and methodically managed as a harmful material. Obviously, the nature of these kinds of wastes is hazardous to human health and exposure to high doses of radiation causes vomiting, nausea, hair loss, diarrhea, hemorrhage, cell and DNA damage, destruction of the intestinal lining and central nervous system damage, increases in the possibility of cancer and even death, as well as contamination of the ecosystem, since it emits radioactive particles, along with causing soil infertility and genetic mutations, seeds to not sprout, slow growth, losses in fertility that can alter attributes of the plant, etc. The radioactive wastes cannot be destroyed, and for this reason, they stay for a prolonged time in the eco-system, escorting a high risk to lives if not correctly managed. Not only this, but the contemporary process of mining uranium (U) discharges elevated quantities of carbon dioxide (CO_2_) into the atmosphere. Additionally, CO_2_ is being emitted into the open air when newer nuclear power plants are constructed and the transport of radioactive waste is being performed. The direct consequences of exposure to ionizing radiation in air, water and food are reported as being very much dodgy. Radioactive wastes may be found in all three forms, i.e., as gas, or liquid or solid, and surprisingly their level of radioactivity also varies. Incredibly, these sorts of wastes may remain at constant levels of radiation for a few hours to several months or even hundreds of thousands of years! 

## 2. Research Methodology

A comprehensive literature review was conducted to identify and appraise allied available information on record, which comprises pedagogic ideas and referenced examples of the fusion work. Recently, one of the rapidly expanding study disciplines in recent years has become a crucial sub-discipline of solidification/stabilization technology for radioactive wastes using cement. To comprehend in-depth the most recent and emerging drift of radioactive waste as edifice material, the keywords “radioactive waste“, “solidification/stabilization technology“, and “solidification/stabilization of radioactive waste using cement”, have been methodically recovered, using bibliographic databases of “Springer”, “Elsevier”, “Taylor and Francis”, “Wiley” and “Hindawi”. Furthermore, comprehensive data analysis and categorization were carried out based on a thorough understanding of titles, graphical abstracts, highlights, abstracts, keywords, entire texts, conclusions, and impressions. The cited literature data represent a comprehensive description of the progress, portrayal, and application of cement in stabilization technology for radioactive waste.

## 3. Classification of Radioactive Wastes

**Figure 2:**
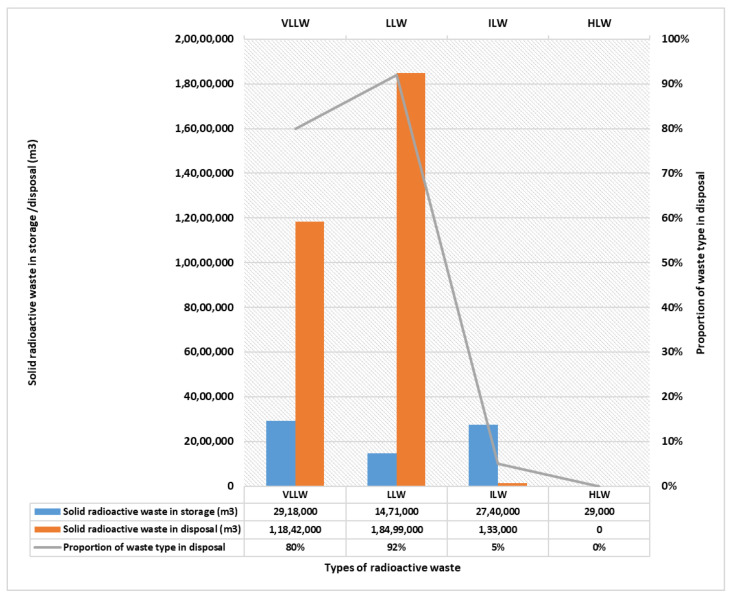
Nuclear waste inventory.

Broadly speaking, the radioactive wastes are classified as low level, e.g., paper, tools, clothing, rags, etc., which enclose petite quantities of chiefly short-lived radioactivity; intermediate-level wastes with elevated amounts of radioactivity, therefore, necessitate some shielding. The LLW and ILW are the wastes generated from general operations such as the cleaning of reactor cooling systems as well as fuel storage ponds, and the decontamination of tools, filters, and metal components get polluted and become out to be radioactive on account of their utilization in or near the reactor. Lastly, the third group comprises high level wastes which are extremely radioactive and hot, owing to decay heat, and hence they need both cooling and shielding. The storage time period of radioactive wastes relies upon the type of waste and radioactive isotopes. The long-term storage of high level wastes requires burial in deep geological formations; however, short-term storage can be implemented on or near to the Earth’s surface. Most low-level and short-lived intermediate level wastes, in general, experience land-based disposal at once after packaging. Mostly, at present, the near-surface disposal facilities are in operation, viz., a few low-level liquid wastes from reprocessing plants are disposed of in the sea, including radionuclides; however, this is regulated and controlled, and the uppermost radiation dose anyone gets from them is a small fraction of natural background radiation only. The long-lived ILW and HLW include spent fuel when regarded as a waste, which stays radioactive and is subjected to deep geological disposal. The safe techniques for the ultimate dumping of high-level radioactive waste are verified technically and the global consensus is “the geological disposal is the most excellent feasible systematic solution”. Significantly, with a view of ultimate dumping, the “multiple barriers” geological disposal is planned to make sure that no noteworthy environmental releases take place for more than tens of thousands of years. This is a valuable method to immobilize the radioactive elements in HLW and long-lived ILW, and to isolate them from the bio-sphere. Notably, nuclear power is the only huge-scale energy-producing technology that has the potential to take complete responsibility for all its radioactive waste and fully encapsulate it into the product. The monetary provisos are planned for the management of every single civilian’s radioactive waste. The cost of management and disposal of nuclear power plant wastes is normally more or less 5% of the total cost of the generated electricity. Figure 2 displays the Nuclear waste inventory for LLW, ILW, HLW, VLLW.

There are three general categories of radioactive wastes:

### 3.1. Low (Including Very Low)—Level Radioactive Wastes

**Figure 3:**
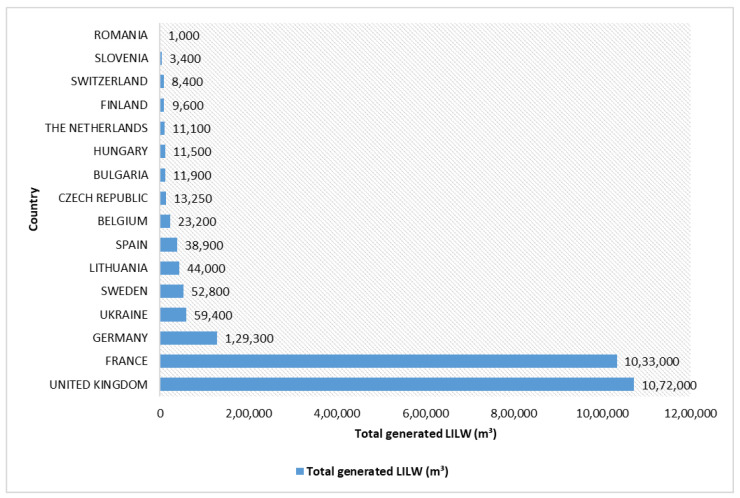
Total generated LILW in different countries.

The exempt waste and very-low-level waste comprises radioactive materials at a harmless level for lives or the ecosystem. For the most part it consists of demolished material such as concrete, bricks, metal, piping, plaster, valves, etc., produced during rehabilitation or dismantling in nuclear industries. As a result of the concentration of natural radioactivity present in definite minerals exercised in their manufacturing, some other industries, namely, food processing, chemical, steel, hospitals, industry, as well as the nuclear fuel cycle, etc., also produce this sort of radioactive wastes. The low-level waste is radioactively contaminated industrial or research waste, i.e., short-lived radioactivity, which includes general items such as paper, protective clothing, plastic bags, cardboard, tools, clothing, filters, and packaging material, etc., when they get in touch with radioactive materials in any industry using radioactive material such as government, manufacturing unit, medical, utility, research facilities, etc. The near-surface disposal of low-level wastes is commonly done since they possess a radioactive content not within the limit of 4 giga-becquerels (GBq) per tonne (GBq/t) of alpha activity or 12 GBq/t beta-gamma activity. For this reason, no shielding is essential during handling and transport. It is fit for dumping in near-surface facilities. It contains roughly 90% of the volume but possesses merely 1% of the radioactivity of all radioactive wastes. The total LILW generated in different countries are presented in Figure 3.

### 3.2. Intermediate-Level Waste

Intermediate-level radioactive waste (ILW) is reported to be more radioactive than low-level radioactive waste (LLW), however, the heat generation is <2 kW/m^3^, which is insufficient to be considered for the design or selection of storage and dumping facilities. The ILW necessitates some protection because of its high levels of radioactivity. Characteristically, the ILW comprises resins, chemical sledges, metal fuel cladding, and the polluted materials from decommissioning of the reactor. The small items and any non-solids may be solidified in bitumen or concrete for dumping. ILW constitutes about 7% of the total volume and possesses 4% of the radioactivity of all radioactive waste.

### 3.3. High-Level Waste

The high-level waste (HLW) is full of extremely radioactive and for the most part comparatively short-lived fission products, creating a concern. If the waste is stored, possibly in deep geological storage, after several years the fission products decay, lessening the radioactivity of the waste. Significantly, the HLW is adequately radioactive for its decay heat of >2 kW/m^3^ to elevate its temperature, as well as the temperature of its surroundings. For these reasons, the HLW needs both cooling and shielding. The radioactive wastes are being produced at each stage of the production of electricity from nuclear materials, i.e., the nuclear fuel cycle, which involves the mining and milling of uranium( U) ore, its processing and fabrication into nuclear fuel, its utilization in the reactor, its reprocessing, the treatment of the utilized fuel coming from the reactor, and the waste dumping. Thus, they arise from the “burning” of uranium (U) fuel in a nuclear reactor and encloses the fission products and trans-uranic elements generated in the core of the reactor when electricity is produced. Statistically, the HLW accounts just for 3% of the total volume; however, it provides 95% of the total radioactivity of produced waste. Highly radioactive fission products and trans-uranic elements come from uranium (U) and plutonium (Pu) during the operations of reactors, and are enclosed inside the spent fuel. When used fuel is not reprocessed, it is regarded as a waste of the HLW type.

There are two different types of HLW, as follows:

- Utilized fuel, which is designated as the waste.
- Separated waste from the reprocessing of utilized fuel.

The HLW has both types of components, i.e., long-lived and short-lived ones, relying upon the length of time period needed to decrease the radioactivity of definite radionuclides to levels that are regarded as safe for the public and the neighboring atmosphere. The difference turns out to be vital for the management and dumping of HLW, if normally short-lived fission products can be separated from long-lived actinides. The HLW is the center of noteworthy attention concerning nuclear power, and is managed in view of that. The HLW includes utilized nuclear fuel from nuclear reactors along with the waste generated from the reprocessing of used up nuclear fuel. The majority of used up nuclear fuel comes from nuclear power plant reactors of the commercial type. At this time, mostly high-level waste is stored at the site itself where the waste is generated.

## 4. Nuclear Power and Defense Operations—The Sources of Radioactive Wastes

**Figure 4:**
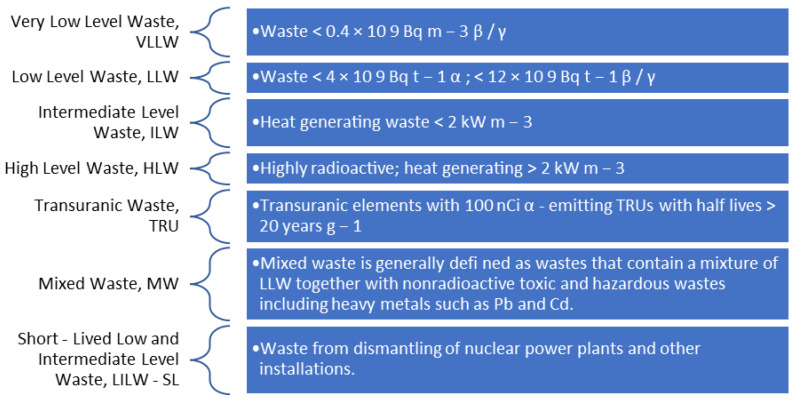
Different Categories of radioactive waste.

Commonly, the radioactive wastes are further divided into numerous precise categories relying upon their activity on the whole as briefed through the Figure 4, i.e., very-low-level waste (VLLW), low-level waste (LLW), intermediate-level waste (ILW) and high-level waste (HLW).

The high-level waste generates heat and hence, the temperature of this waste may rise drastically owing to the result of radioactive decay courses at least in the shorter time-scales. The sources of HLW include high-level liquid waste (HLLW) produced in the duration of the reprocessing of used up nuclear fuel that encloses a lot of short-lived fission products along with actinides and longer-lived fission products. One more source of HLW comes from the production of plutonium (Pu) metal and tritium (H-3 or 3H, or T)—the only radioactive isotope1 of hydrogen, used in weapon applications.

Additionally, the ILW may be heat-generating, however to a lesser degree than HLW, and chiefly comprises items such as the components within nuclear reactors, including graphite from reactor cores, fuel element debris, and fuel cladding along with radioactive sources employed in experimental instruments or medical equipment, chemical sludges and filters, which is defined as waste with an activity of >4 × 10 9 Bq t—1 α—radiation and >12 × 10 9 Bq t—1 β—and γ—radiation. By and large, this sort of waste is encased in concrete inside steel containers and put into storage in anticipation of final disposal. Some other categories of treatments of ILW that are not very heat-generating include pyro-chemical, electro-refining, and associated wastes from the reprocessing of plutonium (Pu) metal for weapon applications, and these also necessitate particular types of treatment. 

The activity of LLWs, including mainly worn shields and a few pieces of equipment or materials employed in the radioactive facilities, along with polluted soil and construction materials and varied organic and inorganic materials, though, comparatively low, cannot be dumped off as ordinary waste. The activity of LLW is set at <4 × 10 9 Bq t—1 α—radiation and <12 × 10 9 Bq t—1 β—and γ—radiation. At present, it is compacted into steel drums which are positioned within the bulky boxes and packed with concrete. The activity is very low in the case of VLLW and hence, it can normally be dumped off as ordinary waste, either in domestic landfills or by undergoing incineration treatment. It is, in general, defined as waste enclosing <0.4 × 10 9 Bq m—3 β—and γ—activity.

In U.S.A., an additional category of radioactive waste is defined for particular wastes with a lower activity than HLW that comprises transuranic elements, called “transuranic waste (TRU)” and encloses 100 nCi of α—emitting transuranic elements with half-lives > 20 years per gram of waste [37]. 

Several supplementary materials such as surplus uranium (U) and plutonium (Pu) from both civilian and military nuclear programs were at one stage throughout the 1990s well thought-out as wastes and may at some future date be affirmed as wastes again, although this is nowadays becoming more and more unlikely. At this time, used up nuclear fuel is being stored with no reprocessing, which is also considered as nuclear waste, although, the newly altering situation, brought about by the rising universal exigency for energy, and especially for energy sources that do not release considerable amounts of greenhouse gases (GHG), meticulously, CO_2_—carbon dioxide—is now leading to an international drive to construct new nuclear power stations, and it will need fuel recycling. In 2008, the UK Government announcement supported principally the construction of new-fangled plants. It is reported that the total quantity of nuclear power generated globally at present is of the order of 370 GW [38], which is estimated to augment between 447 GW and 670 GW by 2030. China and no-one else is setting up to construct 30 brand-new reactors by 2020, whilst India is now structuring seven novel nuclear plants. In Finland, one is under construction at Olkiluoto and in the U.S.A., many states pointed to the awareness of the construction of newer nuclear power stations. It is approximated that three to four novel plants would have to be constructed annually, opening in 2015, just to uphold the existing 20% nuclear power supply share of U.S.A. [39]. The exigency for electricity in U.S.A. is predicted to rise to 30% by 2030. Thirty-five, brand-new reactors are planned to be built. Although the proposed costing of a new-fangled facility is amplified to US$12–18 billion, the people are currently primarily in favour of nuclear power. Consequently, it is very obvious that nuclear power is certainly came back on the agenda, with lots of articles also appearing in the well-liked press stressing this modification in political trends. This is posing a very solemn challenge for scores of nations that are short of a big enough pool of skilled personnel and fresh graduates in the nuclear sciences, counting waste management specialists too. 

Additionally, there also exist a few remarkable types of radioactive wastes. They include the following:

### 4.1. Transuranic Waste (TRUW)

In accordance with the U.S. regulations, Transuranic waste (TRUW) is defined as, with no regard to form or origin, waste that is polluted with alpha-emitting transuranic radionuclides with half-lives beyond 20 years and concentrations more than 100 nCi/g (3.7 MBq/kg), exclusive of HLW. Those elements, which possess an atomic number bigger than uranium, are called transuranic, meaning “beyond uranium”. On account of their long half-lives, TRUW is disposed of with more care than either LLW or ILW. That simply means that the “transuranic wastes” are synthetic radioactive elements possessing an atomic number of 92 (uranium) or higher. In the U.S.A., mostly, transuranic waste is found generated from nuclear weapons productions, which encloses common items, viz., tools, and lab equipment polluted for the duration of the initial age of nuclear weapons research and development. Presently, this kind of waste is stored at federal facilities and finally disposed of. 

### 4.2. Uranium (U) Or Thorium (Th) Mill Tailings

The mill tailings of radioactive wastes stay around following the mining and milling of uranium (U) or thorium (Th) ores. They are stored at the sites of their generation in specifically designed ponds known as “impoundments”.

### 4.3. Technologically Enhanced Naturally-Occurring Radioactive Material (TENORM)

A few radiological materials can subsist naturally in the eco-system. Other sources of radioactive wastes include medical and industrial wastes, as well as naturally occurring radioactive materials (NORM). At times, these naturally-occurring radiological materials (NORM) can turn out to be concentrated in the course of human activities such as mining or extraction of natural resource, the processing or consumption of coal, oil, and gas, and some minerals, bringing coal to the surface or burning it to produce concentrated ash, etc. This concentrated or relocated NORM is called “Technologically Enhanced NORM, or TENORM”, which is generated from plenty of industries and processes such as oil and gas drilling, mining, and production, as well as water treatment. The said waste must be disposed of or managed systematically as per the rules and regulations of authorities. Globally, the highest Tenorm waste stream is found to comprise coal ash, with by and large 280 million tons annually, which includes uranium-238 and all its non-gaseous decay products in addition to thorium-232 and its progeny. More often than not, the referred to ash is just buried, or may be employed as a constituent for construction materials. TENORM is not regulated as limitedly as nuclear reactor waste, although there are no major differences in the radiological perils of these materials.

### 4.4. Legacy Waste

Apart from the routine radioactive waste from current nuclear power generation, there is one more kind of radioactive waste, known as, “legacy waste” that exists in pioneered nuclear power and particularly where power programs were developed out of military programs. At times it is found in volume, creating difficulty for its management. Owing to momentous activities typically associated with uranium mining, military programs, and the radium industry, copious sites enclose or are polluted with radioactivity. Single-handedly in the United States, the Department of Energy reports that there are “millions of gallons of radioactive waste” and “thousands of tons of spent nuclear fuel and material” along with the “huge amounts of polluted soil and water”.

## 5. Impacts of Exposure to Radioactive Wastes

Exposure to radiation from radioactive wastes may cause adverse health impacts due to ionizing radiation, such as:

A dose of 1 sievert carries a 5.5% risk to mankind of developing cancer. 

Ionizing radiation can cause deletions in chromosomes of human beings.

A possible birth defect in children.

May influence DNA, mRNA and protein repair.

The thyroid gland may be injured.

A propensity to have long biological half-lives and a high relative biological effectiveness makes it far more damaging to tissues, etc. 

## 6. Illustrations of Accidents While Dealing with Radioactive Wastes

Unfortunately, a few incidents have taken place in the world’s history where radioactive material was disposed of inappropriately, such as in the case of defective shielding during transport, or due to it simply being abandoned or even stolen from a waste store. The few remarkable accidents are:

- The Goiânia accident, which involved radioactive scrap originating from a hospital.
- In Japan, the nuclear substances were found in the waste of Japanese nuclear facilities.
- The waste stored in Lake Karachay in the old USSR was blown over the region during a dust storm following the lake partially drying out. In a low-level radioactive waste facility located at Maxey Flat, in Kentucky, containment trenches were covered up with dirt, instead of cement or steel, and fell down under the action of heavy rainfall into the trenches and ultimately filled with water, which invaded the trenches and turned out to be radioactive.
- Quite a lot of Italian deposits of radioactive waste have run into river water; therefore, domestically useful water has become polluted.
  Several accidents occurred in France during the summer of 2008. They are:
  (i) At the Areva plant of Tricastin, the liquid enclosing untreated uranium (U) overflowed in a faulty tank during a drain exercise and more or less 75 kg of the radioactive material percolated into the ground and finally into two nearby rivers;
  (ii) More than 100 staff members became contaminated with lower doses of radiation after the deterioration of the nuclear waste site on the Enewetak Atoll in the Marshall Islands and a prospective radioactive spill.

## 7. Radiation Concerns

As illustrated in Figure 4, there exist scores of various public apprehensions in the context of radioactivity and the diverse radioactive waste materials that exist, since the radioactive substances and radiation are being recognized as exceedingly hazardous. To add to this, these concerns also exist with regard to the wastes generated from nuclear power stations that they may get discharged into the eco-system. Of course, radiation is a natural occurrence and can be handled safely by using appropriate protections. Chiefly, uranium (U), thorium (Th) and potassium-40 (^40^K) radioactive isotopes present in the crust of the earth are making basic grounds for natural radiation jointly with their decay products, such as radon gas (Rn), significantly [40]. There exist copious and diverse sources of background ionizing radiation to which each one is generally exposed. The maximum levels take place in geological regions whereby granitoid rocks or mineralized sands are found predominantly on account of traces of natural radioactive materials such as minerals enclosing U and Th as well as Rn (radon gas)—a decay product. Additionally, living at higher altitudes escalates the cosmic radiation level received. The applications of the X-rays in medical and dental can also boost exposure. The background radiation consists of roughly 87% of total natural radiation in association with 13% from synthetic resources. In order of severity, the radiation comes from medicinal applications, an assortment of varied sources, fallout from early atmospheric nuclear weapons examinations, job-related exposure, and radiation caused owing to nuclear discharges. At this juncture, a note should be made that there do not exist differences in the effects on materials among natural and non-natural radiation, including bio-systems. The effects of radiation on human beings are gauged in an international (SI) unit known as the Sievert, symbolized as Sv, which relates to the absorbed dose in human tissue to the effective bio-damage of the radiation. Sievert (Sv) is a unit of the effective dose received (1 Sv = 100 rem, i.e., Roentgen Equivalent Man (rem); or we can say, 1 rem = 0.01 Sv). It is well-known that the smallest dose received by each person breathing on the planet earth consists of a typical natural background level of approximately 2 mSv per year. There does not exist any scientific evidence of risk such as the development of cancers, etc., at doses less than 50 mSv per year in the short-term or 100 mSv per annum in the long-term. Nevertheless, it is also identified that a short-term dose of more than 1000 mSv is regarded as a great enough dose to cause instant radiation illness, whereas a dose of 10,000 mSv by and large results in death within a few weeks only.

## 8. Interventions with Hydration of Cement

The vast varieties of industrial courses of actions can generate wastes, which may get treated by stabilization/solidification (S/S). Of these, the inorganic wastes have a propensity to be more well-suited with cementitious binders. For the most part, the wastes treated by S/S with cement enclose metallic pollutants in an inorganic matrix made up largely of calcium (Ca), aluminum (Al), and silicon (Si), viz., dusts from air-contamination control systems, sludge, and soils. The USEPA declared that organic compounds interfere with cement-based S/S, in particular when the organic concentration goes beyond 1% of total organic carbon (C) by mass. Nevertheless, there exist several illustrations whereby the organic wastes have hardened with cement [41]. Numerous inorganic wastes enclose a few organic stains. In point of fact however that a few wastes can productively be incorporated or treated with cement in more elevated quantities than others. Virtually, there are no limitations on their chemical and physical attributes, other than that they must be finely divided solids or liquids at ambient temperature. The wastes possibly will enclose small additions of chemicals such as CaCl_2_ or gypsum, which can be used as supplements and admixtures in traditional cement and concrete utilizations, but the wastes may also hold lots of dissimilar compounds that would not otherwise be incorporated with cement, including destructive pollutants. Each and every waste components can be coined as “impurities”. This means that they are potentially reactive materials, which would not customarily be present in industrial cement. In the case of S/S, the interaction of wastes with binders is of much importance from two key points of views:

1. The interferences of impurities with hydration of cement together with setting and strength development, as well as matrix durability;
2. Immobilization of pollutants.

The referred to features are correlated in the sense that the development of a well-built durable matrix of inferior permeability is more often than not significant for the immobilization of pollutants, and is consequently a target of S/S treatment. Both beneficial and detrimental interactions can take place among wastes and hydraulic binders, i.e., “cement”.

Still, the comprehensive progressions of hydration of cement stay as a topic of rigorous study and are at variance for dissimilar kinds of cement. Generally, it advances by dissolution of the phases of anhydrous cement from the surface of the particles of cement in the mixing water, followed by precipitation of products of hydration to build a strong cohesive matrix. The impurities can modify the customary hydration of cement at diverse phases; consequently, the dissimilar compounds have unlike, and at times multiple, interaction mechanisms with the binder. The impurities can be modified through altering the chemical composition of the developing pore solution in context of the pH, ionic strength, and chemistry:

- Solubility of anhydrous stages;
- Dissolution kinetics of anhydrous stages;
- Rate of development of yields of hydration;
- Rate of nucleation of yields of hydration;
- Morphology of the hydration yields;
- Chemistry of the hydration yields.

Often, the referred to influences rely on concentration and can differ in accordance with the conditions of curing. While impurities with different interference mechanisms as pure compounds are united, as what probably happens in both waste applications and solidification utilizations, the overall influence can be tricky to forecast [42]. Both the setting and hardening are physical demonstrations of the chemical course of action of hydration of cements. The setting is defined by Taylor [43] as “hardening with no noteworthy improvement in compressive strength,” and stiffening as “momentous growth of compressive strength.” Accordingly, interfering with the hydration of cement might result in the mass effects as follows:

- The acceleration/activation of setting or solidifying, counting, or flash setting, whereby the matrix loses its plasticity instantaneously upon the mixing;
- The false setting, whereby the plasticity of matrix is lost swiftly upon the mixing; however, it can be recovered by supplementary mixing;
- An exigency of altered water;
- The setting or hardening retardation, counting, and absolute inhibition of hydration;
- The modified strength growth counting disruption of matrix;
- The changed chemistry of the pore solution.

A speeding up of setting and false setting can create challenges in the cement’s handling, considering the usage point of view, and can promote equipment failure as well as pitiable workability. The demand for increased water can cause an increase in the speed of setting or false setting, or change rheological attributes of the mixture, which can result in a more permeable structure and reduced durability. Often, the modified strength growth is considered as taking the form of enhanced or reduced early strength, however, later strength can also be influenced, and even a yield that seems to set and achieve strength generally at first can deteriorate swiftly afterwards. Several illustrations of interactions of impurities with cement, in either cement and concrete yields or S/S, can be found in the literature review [44,45,46,47,48,49,50,51], which is abridged in the following sections, for all three types of compounds, i.e., organic compounds, inorganic compounds, and other impurities. The referred to outcomes were generated by experimental studies making the use of pure compounds. Additionally, there are a number of research investigations accessible in the literature conducted in order to treat real wastes. It is much more complicated to simplify real wastes because of their heterogeneity and complex structure and chemistry as well. In the literature studies, plenty of investigations are based on the physical demonstrations of setting and strength growth, or at times, on the extents of the heat evolved by hydration reactions. At the simplest level, the retardation reduces the amounts of the hydration yields at a given time, while the acceleration escalates them. Nevertheless, the impurities present in the pastes of cement may also modify the proportions of the hydration yields, alter the ratio of Ca/Si in the C-S-H, create solid solutions with the hydration yields, or craft completely brand-new hydration yields. Quite a few current investigations revealed the influences of wastes and pollutants on leachate pH and S/S product acid neutralization capability; these are suggestive of modifications to the hydration yields [48,49]. These kinds of alterations may bring about vital consequences for the immobilization of pollutants by all of the mechanisms; however, this is a characteristic of S/S with limited studies. Thus, it is obvious that often cements respond radically to small supplements of impurities; it is feasible to employ additives and admixtures to regulate the hydration properties of an S/S yield to trim down the unwanted influences of components present in the wastes.

### 8.1. Influences of Inorganic Impurities on Hydration of Cement

The influences monitored for in several compounds enclosing inorganic and heavy metal components during the hydration of cement are explained in the literature [50,51,52]. The terms of “acceleration” and “retardation” express influences on setting, hardening, or both, as they are applied in the literature in the same fashion. The previous research data reveal the preponderance of studies on Portland cement in the literature, although, at times, the same is also correct for other kind of cement, or incorporated cement systems, e.g., the metal cations of zinc (Zn^+2^), lead (Pb^+2^), cadmium (Cd^+2^), and chromium (Cr^+2^) also shrink the strength of geopolymer slag cement [53]. The little influences are talked about in-depth in the following notes that refer to the superscripts in the tables:

- The C3A and C3S are accelerators, and they also have a propensity to be the center of attention of action by other accelerators and retarders. These are the uppermost reactive phases in Portland cement, and also most imperative for setting and early strength growth. The other reactive calcium aluminates or Ca–aluminate cements are also accelerators for Portland cement, and vice versa. The referred to species play the role of accelerators or activators for pozzolans. Additionally, lime or cement kiln dust can be employed as activators for pozzolans.
- The carbonates of alkali demonstrate the surprising nature of the few compounds that interfere with the cement setting. The small proportions of less than 0.1% of alkali carbonates were found to retard the Portland cement setting; an augmented quantity results in flash setting, and further boosted quantities can have no influence on setting, while flash setting takes place at very elevated proportions. So far, the interference mechanism is not well-comprehended, however, it is put forward that the influence of carbonates is owing in part to the production of thaumasite—a calcium silicate mineral, rather than ettringite—a hydrous calcium aluminum sulphate mineral.
- Generally, the salts of potassium (K) and sodium (Na) are believed to increase the pH and cause precipitation of amorphous CH that interferes with C3A hydration, and, up until now, a lot of alkali salts play the role of accelerators of Portland cement. They can be supplemented as activators to cement enclosing pozzolans, whereby they boost the solubility of the anhydrated phases. A few salts, namely sodium chloride (NaCl), accelerate at lower concentrations, though retard at very elevated concentrations.
- Sodium silicate (Na_2_SiO_4_) is a well-liked additive for waste solidification. It is useful as an accelerator or activator for pozzolans, or to devour surplus water as it extends both silicate and a higher pH. In the case of the latter, or in the incident that excessive quantities are added on, silica gel development may promote a physically unstable matrix, with shrinkage and swelling due to the humidity modifications of the nearby environment.
- Sulphates have quite a lot of probable influences on the hydration of Portland cement. Perhaps owing to acceleration or retardation by reaction with C3A and C4AF, they may also lead to false or flash setting, by formulating gypsum in place of ettringite, or by matrix destruction via late ettringite development and having a bulk volume on account of its waters of hydration. Additionally, thiosulphate is accounted as an accelerator. The salts of chloride can also outline enormous chloro-aluminates, which are harmful to the matrix if their formulation is late. Both chlorides and sulphates, as well as carbonates and other anions, can destruct the matrix, provided the solubility of one of their salts in the pore solution is high and crystallization takes place [54]
- An increase in MgO by a couple of percentage points can destroy the cement matrix through gradually hydrating to more bulky Mg(OH)_2_; MgSO_4_ also reacts to give rise to more voluminous products, gypsum and Mg(OH)_2_, as well as degrading C-S-H.
- Evolution of gas can cause matrix destruction, for example, from the reaction of aluminum metal or ammonia at a higher pH.
- While soluble chromium (Cr) salts speed up the hydration of Portland cement, chromium (Cr^+3^) oxide has a modest influence on setting. A review of Mattus [44] suggested that chromium (Cr) replaces silicon (Si) in C-S-H, however, the ultimate strength of the matrix is slimmed down. Chromate, (CrO_4_-^2^) is believed to play a similar role to sulphate and develop chromo-aluminate crystals which cover C3A grains. Like sulphate, chromate can also play as an accelerator.
- The influence of ZnSO_4_ exemplifies the significance of taking into consideration the collective effect of both the anion and cation. While, in general, most Zn-compounds are believed to be retarders of Portland cement. ZnSO_4_ is found to be an accelerator at concentrations < 2.5% and a retarder at concentrations between 2.5% and 5.5%; it entirely slows down the hydration of cement at elevated concentrations.
- Boric acid has a strong accelerating impact on the setting of Portland cement, however, it retards solidifying. The carbonates, hydroxides, aluminates and silicates accelerate setting, but retard solidifying or leave it unaltered; nitrates and halides accelerate both the setting and solidifying. With a view to simplify the outcomes of diverse examinations, we separated out the influences of cations and anions, and searched for interaction impacts; data from twelve literature studies of pure compounds added to Portland cement paste were gathered and utilized to build neural network models of Unconfined Compressive Strength (UCS) as a function of mixture chemistry [55]. The utilization of the most excellent neural network using other information proposed that Cs is a retarder and Cr^+4^ has no influence. The accessible information is distinguished for Hg, K, Mn, Na, and SO_4_^−2^. Impacts were monitored for in the literature for Ca–aluminate cements [56,57,58,59,60,61,62], which are normally lesser utilized in waste management because of the better-quality characteristics of cements based on calcium silicate.
- The literature suggests that Li-salts, with the only exception of Li_2_BO_2_, are the strongest accelerators for Ca-aluminate cements of all kinds and are competent enough to cause flash setting. Li-salts are believed to play a role by creating nucleation substrates for the hydration yields.
- Nevertheless, for the other anions and cations, the wide-ranging impacts on Ca–aluminate cement are accounted for. Quite a lot of researchers have attempted to rank the influences of a variety of cations and anions without harmony among themselves [60]. Some others have given general statements to explain this behavior, e.g., Parker [63] explained that alkalis are accelerators and acids are retarders of Ca–aluminate cement, however, sulfuric acid is well-known to be as an accelerator; Sharp et al. [64] proposed that those compounds which augment the C/A ratio play the role of accelerators, however, CaCl_2_ is well-known retarder.
- The probable variety of the influences is depicted by monitoring for Ca–sulphate [65] and they found gypsum with no impact on the setting time of Ca–aluminate cement, while hemihydrate is an accelerator and anhydrite is a retarder. They proposed that the relations among the dissolution kinetics of the referred to diverse forms of calcium sulphate control both the kinetics and the hydration yields.
- The suggestion has been that CH plays a role as an accelerator for calcium aluminates by boosting the C/A ratio of the solution, and driving precipitation of the calcium-rich phases C2AH8 and C4AH13. The Portland cement is believed to be an accelerator since hydration of C2S and C3S generates CH. Yet again, the neural network analysis was employed to construct models of setting time as a function of mixture composition, utilizing available information for pure compound additions to Ca–aluminate cements. This evealed that the reproducibility of setting time measurement is pitiable, and that this accounts for a few of the differences in the outcomes in the literature.

### 8.2. Influences of Organic Impurities on Hydration of Cement

So far, numerous organic compounds are supplemented as purposeful admixtures to cement and concrete. Their selection and effects are derived from Massazza and Testolin [47]. Some other organic compounds are found usually in waste, such as oil and grease, chelating agents such as ethylene diamine tetraacetate (EDTA), and chlorinated hydrocarbons—trichlorobenzene, phenols, alcohols, carbonyls and glycols. The impacts of organic compounds on the hydration of Portland cement are changeable and extremely concentration-reliant, e.g., generally sugars and triethanolamine are retarders for C3S hydration, but can play the role as both retarders and accelerators for C3A hydration, and will promote flash setting at higher concentrations [44]. The polar solvents hinder hydration to a much larger extent than non-polar solvents as accounted for by Nestle et al. [66]. The findings for definite pollutants were examined by them as well as others also. Additionally, scores of organic compounds result in the progressive worsening of cement yields over time. The majority of organic compounds are retarders of Ca–aluminate cements. Particularly, citrate salts are usually employed as retarders. Nevertheless, Bier et al. [67] presented a technique of utilizing Li and citrate jointly to optimize the setting attributes of Ca–aluminate cements, such that retardation essentially did not occur, while Baker and Banfill [68] utilized trilithium citrate as an accelerator. The trilithium citrate was found to have more or less no effect in experiments carried out by Damidot et al. [56]. Even though pozzolans are accelerators of C3S hydration, they themselves hydrate, and pozzolanic cements are inclined to achieve strength more slowly than Portland cement, unless an activator, viz., an alkali or CH, is employed. Pozzolans may reduce the impacts of other impurities [69] and are well-known to diminish the hydraulic conductivity and enhance the strength and durability of the ultimate yield in the long duration. While an addition is made to Ca–aluminate cements, pozzolans lend a hand to formulate C2ASH8 that resists conversion to lesser voluminous C3AH6 [70,71]. At last, the cement hydration is also influenced by the physical characteristics of the cement and any impurities, i.e., crystal defects, crystallinity, as well as the size of particle and surface area of the dissimilar mineral phases. The reactivity of binders is augmented with fineness, however, finer impurities such as colloidal matter or clay can retard the setting.

## 9. Solidification of Strontium (SR) through Diverse Kinds of Cements

Looking into earlier studies, the research investigations have thrown light chiefly on the leaching of radionuclides in PC-based systems with supplement of diverse minerals such as cement systems based on bentonite, zeolite, and tobermorite [72,73,74]. The radionuclides’ stability in the matrices is found to be improved when the integration of minerals is made. The minerals are chosen on account of their higher adsorption competences. The higher concentration of salts in the LLW and ILW radioactive waste liquid has an immense impact upon the hydration process, hydration yields, rheology behavior, and setting time of cement paste. For these reasons, there are a few issues for the solidification of radionuclides from nuclear plants. Consequently, the leaching rate of radionuclides becomes modified with the product performance and the pore-structure of the matrices [11,75,76,77,78,79,80,81,82,83,84,85,86,87]. Sodium hydroxide’s (NaOH) influence on the structure of toughened Portland cement matrices and the leachability of Sr^+2^ was studied by Zheng et al. [12]. The outcomes pointed towards the improvement in the adsorption competence of C–S–H gel by the use of NaOH and mitigated the porosity of matrices that lead to the decline in the leaching rate of Sr^+2^. Conversely, sodium nitrate (NaNO_3_) boosted the increase in the leaching rate of Sr^+2^ and the adsorption capability of C-S-H gel was also found to be enhanced since the porosity get improved.

Apart from PC, the magnesium phosphate (MgSO_4_) cement (MPC) is considered as a chemically bonded ceramic with good solidification performance for both LLW and ILW liquids.. The application of MPC in the interest to solidify Pu-contaminated ash where the mass quantity of Pu is up to 5% in form of PuO2 was performed in an early research study by Wagh et al. [77]. The mechanism of immobilization comprises the physical encapsulation in the denser matrix together with the lower solubility of PuO2. 

The sulpho–aluminate cement possesses the potential to immobilize Sr^+2^ and Cs^+^ owing to its good retention capability and resistance to salts. Xu et al. [78] have concentrated on the mechanism to immobilize Sr^+2^ and Cs^+^ in the form of radioactive ion exchange resin by the sulpho–aluminate cement. The results of experiments revealed that the competence of immobilization with regard to Sr^+2^ and Cs^+^ by sulpho–aluminate cement is superior to PC. In context of the stages in cement, ettringite can proficiently absorb Sr^+2^ (88%) and Cs^+^ (70%) when the ratios of retention of the aluminum hydroxide gel for the two ions were merely 10% and 40%, correspondingly. The Sr^+2^ immobilization by ettringite is assigned to the substitution of Ca^+2^ in crystal lattice by Sr^+2^.

Type 1 Standard Portland cement (PC), is general-purpose cement fit for all applications where the particular characteristics of other kinds are not essential. Often, this kind is most useful for S/S systems.

Type II Moderately Resistant to Sulphate attack PC is employed where preventative measures against reasonable sulphate attack is vital.

The resistance to sulphate attack is achieved by forming a cement with a lower enclosure of tricalcium aluminate, with the highest proportion reaching up to 8%. Generally, the Type II cement will achieve strength and produce heat at a rate inferior to Type I. In S/S utilizations this might be considered, because, here, there is an involvement of volatile organics. The low temperature of the S/S mixture might reduce the discharge of organic species with a volatile nature.

Type III High Initial Strength PC offers more initial strengths than Type I or Type II, although the eventual long-standing strengths are almost analogous. Both chemically and physically, it is alike to Type I cement, except that its particles are grounded finer. Though rich mixtures of Type I cement can be employed to obtain increased early strength, Type III cement might offer this more agreeably and more cost-effectively. The swifter hydration will normally discharge heat more rapidly and grounds for a somewhat higher rise in temperature than Type I.

Type IV Lower Hydration Heat PC is utilized where the quantity of heat produced should be minimized as found in the case of great structures of concrete, viz., huge foundations and dams. However, accessibility is tremendously restricted mostly since parallel properties can be attained from a Type I cement, more often than not comprising of a mix of Type I cement and fly ash (FA). Since it lacks in accessibility and fitting substitution, Type IV cement perhaps has low utilization in S/S applications.

Type V Higher Resistant to Sulphate attack PC is utilized where S/S systems either enclose or are subjected to rigorous sulphate action—chiefly whereby soils, waste, or sub-surface water own higher quantities of sulphate. It obtains strength more leisurely than Type I or Type II cement. On account of its low content (highest up to 5%) of tricalcium aluminate, it resists more against sulphate attack than Type II cement. By and large, the amount of iron is greater in Type V cement, and this might be sought after if the species present in the waste form are insoluble iron complexes.

The magnesium–potassium phosphate cement (MKPC) is an efficient agent for S/S technology. Although MKPC demonstrated optimistic impacts on S/S, some studies were carried out on the mechanism of the S/S of heavy metals by MKPC. This was in accordance with the suggestion of Singh et al. [79], who projected that the cause for the immobilization of chemically bonded phosphate ceramics is chemical stabilization, the reaction kinetics among pollutant metal salts and the phosphate solution, followed by physical encapsulation inside the denser matrix of phosphate. The particles of fly ash were encapsulated in the binder matrix as found by Rao et al. [86]. In the context of the hydration yields of heavy metal, Jeong et al. [81] unearthed that PbO can structure the PbHPO4 in magnesium phosphate cements (MPCs).

The MKPCs solidify cement paste powders produced from the heating and grinding of radioactive concrete wastes. The benefits of the application of MKPCs are their higher early strength, higher bonding strength, little drying shrinkage, lower permeability, and higher resistance to sulphate attack [82,83,84]. Quite a lot of investigations on the applications of MKPCs for the solidification and stabilization of radioactive or heavy metal wastes have been conducted [85,86,87]. The compressive strengths of MKPC waste forms are reported to be just about two-fold greater than the ordinary Portland cement (OPC) grouts, and the porosities are mitigated by half [83]. Additionally, the MKPCs display a greater resistance against radioactivity in comparison with PC [88,89], authenticating the potential of MKPCs for radioactive waste solidification. To add to this, unlike vitrification or ceramic solidification procedures, MKPCs are cost-effective and energy-proficient and employ uncomplicated equipment; particularly, there is an absence of volatilization of nuclides.

On the other hand, MPCs are employed to stabilize and solidify an assortment of lower level radioactive mixed wastes, Pu-polluted ashes, and technetium enclosing waste solutions at the Argonne National Laboratory, USA [83,90]. The stabilization of a higher content of sodium waste streams with MPC is studied by Colorado et al. [91]. Vinokurov et al. [92] investigated the immobilization of simulated liquid alkaline high-level waste (HLW) enclosing actinides and fission as well as corrosion yields with magnesium (Mg)–potassium(K)–phosphate (PO_4_) matrices. Therefore, the viability of MPC for the S/S technology for risky and radioactive wastes is verified by prior studies.

Calcium Aluminate Cement (Cac) and Cac Modified With Phosphate (Cap) cements are some of the substitute cements in PC and are renowned materials, which are exercised for solidification and for the encapsulation of radioactive wastes [90]. Usually, it is incorporated with supplementary cementitious materials (SCMs) such as fly ash or blast furnace slag. The CAC is characterized by the internal environment possessing a considerably lower pH, ranging from 10.5 to 1,1 than that of PC and its integrated systems, displaying more than 13 [93]. The encapsulation of the reactive metallic radioactive wastes in CAC is examined through making the use of this property. The alteration of CAC with phosphate (CAP) can lower the pH in a range of 9.0 to 10.5 [93]. Additionally, the alteration of CAC by phosphate is identified to stop the traditional hydration of CAC, and results in solidification through an acid–base reaction amongst acidic phosphate solution and CAC powders playing the role as a base [94,95,96,97,98,99,100]. Recently, Caps have been examined for their use in S/S technology of dangerous as well as radioactive materials, because of their capabilities of solidification reactions and/or internal environment pH lowering [101,102,103,104,105].

## 10. Impact of Cement Solidification Technology

### 10.1. Compressive Strength

In a study by Laili et al. [106] on the solidification of radioactive waste resins, they utilized cement mixed with dissimilar percentages (0%, 5%, 8%, 11%, 14% and 18 %) of organic material, called “biochar”. The outcomes demonstrated that the biochar content had an influence on the compressive strength of the solidified resins. The outcomes for the effect of biochar dosage displayed that the addition of 14 wt % of biochar augments the value of compressive strength from 6.2 MPa (5 wt %) to 10.1 MPa. Further than this percentage, the compressive strength was found to be somewhat diminished to 9.2 MPa (18 wt %). Biochar can absorb water and this absorbed water in biochar does not bind chemically with carbon. As a result, the absorbed water would be discharged all throughout the hydration process, which might support the hydration procedure during the initial age of cemented waste. The competence of water retention in biochar may cut the water loss through evaporation that offers good conditions for curing designed cemented waste.

Nishi and Natsuda [107,108] worked on an improvement in resins load and compressive strength of solidified waste and pointed out that resin loading for solidification into OPC must be limited to lesser than 20% to put a stop to the development of cracks at elevated loadings that will result in an unsteady waste yield. The cracks of the cement waste form are assigned to the produced swelling pressure of resin of 50 MPa (max)), which goes beyond the tensile strength of the cement waste form. Pan [109] investigated resin encapsulation using furnace slag and fly ash as admixtures and pointed out that a loading of 24 wt. % of resin can be encapsulated when the use of a combination of furnace slag (24 wt. %) and fly ash (24 wt. %), and OPC (8 wt. %) is made. The supplementation of zeolite into OPC in order to be encapsulated into the resins was focused on by Bagosi [110], who designated that 24 mL resin can be encapsulated into a mix of 55.9 g OPC, and 37.3 g zeolite. Supposedly, the weight of 24 mL resins is 40 g, then the proportion of resin loading is more or less 43%. With a view to boost the resin loading and stability of the waste products, fibers of stainless steel, glass or carbon can be supplemented. The upshots [111] unearthed that the addition of definite fibers enhanced the stability of the solidified waste product, although the fibers of glass and carbon are not found fitting for resin solidification into cement because fibers of glass are not alkali-resistant and get dissolved in the cement matrix. One challenge with the carbon fiber application is the formation of a homogenous waste form. At this time, Durafiber or polypropylene is exercised in the cement industry to minimize the cracks in the cement structures, and may be regarded for the encapsulation of spent radioactive resins provided this fiber is radiation-resistant.

Natsuda [108,109,110,111,112] conducted research with a view to enhance resin load and compressive strength of the solidification products. In a final conclusion, it has been reported that the resin load in the solidification products of OPC must be regularized at lower than 20% volume of wet resins to solidification product. While resins load is greater than 20%, the cracking is engendered often and the solidification products display inferior stability. Additionally, the resins’ expansion in water was studied, and it was reported that the expansion of resins is one of the key causes which results in cracking. The expansion pressure range is reported to be between 0 to 50 MPa. The resins must be saturated in water previous to solidification with a view to avoid cracking. An expansion model was set up, and from this model, when capabilities for ions and water of resins achieve saturation, the expansion pressure must be zero. There must be no cracking and/or fractures, however, the experiments exhibited that when resins loads are greater, there would be the development of fractures in the solidification products even though the resins are saturated.

### 10.2. Behavior of Leaching Process

“Leaching” is a procedure through which a liquid dissolves and takes away the soluble constituents of a material. At times, “leaching” occurs by means of percolation or the flow of water, which can smash up cement-based structures rigorously, e.g., dams, pipes, conduits, etc. It can potentially degrade the cement-based engineered barriers employed for long-standing storage of nuclear wastes.

Simply speaking, the diffusion of radioactive ions into outer medium through conjoint pores is known as “leaching”. The rate of leaching of pollutants is confirmed by the characteristics of the waste form, such as the pore structure, quantity of water, hydraulic conductivity, and homogeneity. Numerous research studies are conducted on the relations among ion leaching and the micro-structure of the solidification yields. The researchers have found that minimizing pore size in solidification products and enhancing pore structures are the efficient and valuable techniques in the interest to mechanically enclose wastes in solids. Hence, a lower ratio of water/cement (W/C) and compressive molding are exercised for minimizing the pores in solidification product, and surface painting is also utilized to clog pores in the products.

The release rates are estimated for quite a lot of radionuclides such as Cs-134, Mn-54, Co-60, Zn-65 and Eu-152. The release data designate that merely one of the formations exhibit the leaching of Cs-134 from the hardened spent resins. Additionally, there are no other radionuclides such as Mn-54, Co-60, Zn-65 and Eu-152 spotted in any leachates. The bulk of research carried out on leaching by world researchers has utilized a much higher total activity of radionuclides. For instance, Rudin et al. [113] have spiked 75 μL of a NIST-perceptible SeCl2, with Se-75 having a total activity of 19,443 Bq in the paste of cement. The samples taken for use for the leaching test by Papadokostaki and Savidou [114] possessed the total activity of roughly 370 KBq of Cs-137. Though the early activity of radionuclides in spent resins is regarded as low the waste form produced in the said research studies appears to be capable of immobilizing the radionuclides [115]. Accordingly, the findings put forward that further research of leaching tests utilizing spiked solutions with a higher total activity of radionuclides into fresh ion exchange resins must be carried out.

Interestingly, Li [116] monitored the rate of leaching of nuclides. The dumping of spent ion exchange resins was performed subsequent to the encapsulation into cement. The heavy metals were then precipitated in the matrix of cement whereas alkali metals, namely, cesium (Cs), stayed considerably soluble and leach out from the waste form under conditions of cement encapsulation. The findings designated that even though Cs-loaded resins are encapsulated, the matrix will have a leaching rate that is one or two orders of magnitude more in comparison with the leaching rate for Cs from the resins themselves. The recent research concentrates on the diminution of Cs leaching in terms of the total Cs adsorbed on the resin through amalgamation of natural and chemically treated zeolites into the cement as an admixture. The study results indicate that the supplement of natural zeolites slimmed down the Cs discharge to around 70% to 75% of the amount initially bound in the resin over a leaching period of three years. Kaolin clay has an influence on the leaching attributes and strength of the cemented waste form. The results of leaching examinations uncovered that the addition of kaolin into cement mitigates the rates of leaching of radionuclides considerably. Nevertheless, the outcomes unveiled that supplement of clay in a surplus of 15 wt. % trims down the hydrolytic stability of the cemented waste form. The lowest rate of leaching and highest strength were recorded when the addition of 5 % kaolin content to the cement matrix was made.

Favorably, the exhausted ion exchange resins are cemented for dumping. The heavy metals precipitate readily in the higher pH environment of cements, however, the alkali metals such as cesium (Cs) stay significantly soluble. The cementation of Cs-loaded resins has setbacks because when they cemented, they display rates of leaching that are one or two orders of magnitude more elevated in the cement matrix than in the resins themselves. The investigations by Bagosi [117] have thrown lights on the diminution of noteworthy Cs leaching in terms of the total Cs adsorbed on the resin through adding natural untreated and chemically treated zeolites to the cement. Finally, they concluded that the supplement of natural zeolites declined Cs discharge by up to 70% to 75% of the amount first bound in the resin during the course of a leaching period of 3 years. Osmanlioglu [118] supplemented kaolin clay into cement with a view to bring down the rates of leaching. The influences of kaolin clay on the leaching characteristics of the cemented waste forms were evaluated, and the impact of the kaolin addition on the strength of the cemented waste form was also tested. Notably, the long-lasting examinations of leaching put on show that kaolin addition into the cement mitigates the rates of leaching of the radionuclides. Nevertheless, the supplements of clay in surplus of 15 wt.% were found to cause an imperative dwindle in the hydrolytic stability of cemented waste forms. Furthermore, it is reported that paramount waste isolation, sans causing a loss in the mechanical strength, was achieved when the kaolin content in cement is 5%. El-Kamash [119] worked on the leaching of 137Cs and 60Co radionuclides fixed in cement and cement-based materials and found that the leaching examinations of 137Cs and 60Co radionuclides symbolize the behavior of leaching and a few of the archetypal radionuclides detected in lower level solid waste forms. The addition of 0 to 15% silica fumes and ilmenite to cement resulted in a reduction in the rate of leaching of each nuclide at diverse studied temperatures. The examinations were carried out for a research study on the leaching of heavy metal ions from cementitious waste. A variety of mathematical models were then employed to gauge the behavior of embedded radioactive wastes. An iterative model was suggested by Krishnamoorthy et al. [120] in order to simulate the rates of discharge of radionuclides from cylindrical-shaped blocks of cement. Two expressions of the leach rate for the diffusive discharge of radioactive components from both cylindrical- and rectangular-shaped waste forms are derived by Pescatore [117]. As an ensemble view, our collaborators managed to use alternative materials for radiation shielding and as well as utilize wastes towards obtaining sustainable materials [121,122,123,124,125,126,127].

## 11. Traditional Treatment of Organic Liquid Radioactive Waste

**Figure 5:**
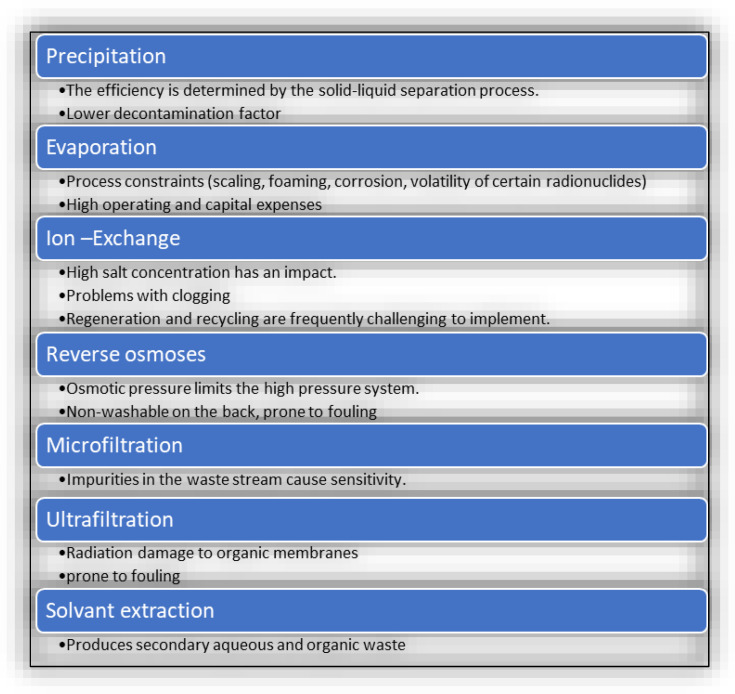
The limitations of various aqueous liquid treatment solutions.

Effluents from nuclear power plants and some medical research institutes contain radioactive heavy metals as well as complex combinations of dangerous organic chemicals and irradiated surfactants. In comparison to other types of radioactive waste, the volume of organic liquid radioactive waste created is minimal. Figure 5 lists typical organic waste kinds, sources, and characteristics. Figure 5 also depicts the characteristics and limits of several technologies used in the treatment of organic liquid wastes. Nuclear power plant liquid radioactive waste typically comprises soluble and insoluble radioactive components (fission and corrosion products) as well as nonradioactive chemicals. The overarching goal of waste treatment procedures is to disinfect liquid waste to the point that the decontaminated bulk volume of aqueous waste may be discharged into the environment or recycled. The waste concentrate is further processed, stored, and disposed of. Because nuclear power facilities create practically every type of liquid waste, nearly every procedure is used to treat radioactive effluents. Liquid waste streams are frequently decontaminated using standard procedures. Each procedure has a distinct impact on the liquid’s radioactive concentration. The extent to which they are employed together is determined by the amount and source of contamination. Evaporation, chemical precipitation/flocculation, solid-phase separation, and ion exchange are the four basic technological procedures for treating liquid waste. These therapy methods are well-known and frequently used. Nonetheless, several nations are making attempts to increase safety and economy via the use of modern technology. Evaporation achieves the best volume reduction impact when compared to the other procedures. Decontamination factors ranging from 10^4^ to 10^6^ are achieved depending on the content of the liquid effluents and the kind of evaporators. 

## 12. Conclusions

The safer and systematic disposal of radioactive waste is of the highest precedence and, hence, the development of secure engineered barriers requires greater focus and attention. The cementitious binders for the use of radioactive waste immobilization offer a solution, which is not only stable but lucrative. The key researchers of resins solidification are concentrated on escalating the loading of spent resins, mitigating the leaching of nuclides, and enhancing the compressive strength of the matrix. The chemistry of Ca–sulpho–aluminate cement is a promising option for the cementation of radioactive spent resins. This kind of cement production is characterized by a lower leaching rate of nuclides, higher spent resin loadings, and stability through wet and dry curing, together with higher compressive strength, and negligible hurdles in manufacturing. The biochar content and spent resin loading affect the compressive strength of the waste form. The review suggests that the magnesium potassium phosphate cements can be utilized to immobilize radioactive concrete wastes generated during the decommissioning of nuclear power plants. The magnesium phosphate cement is competent enough to obtain the rapid solidification of higher content and high-level liquid wastes and radioactive substances in nuclear emergency incidents. The radioactive ions of higher content and high-level can be solidified chemically and encapsulated by the magnesium phosphate cement matrix physically. The potential research in the context of solidification desirably would be concentrated on the control of costs,, solidification competence of perilous components, the optimization of mechanical attributes, examinations of the action mechanisms of diverse components in solid waste, and long-standing stability explorations.

## Data Availability

Not applicable.

## Abbreviations

SCM	Supplementary cementitious material
PC	Portland cement
S/S	Solidification/stabilization
CO_2_	Carbon Dioxide
IAEA	International Atomic Energy Agency
LLW	low-level radioactive waste (LLW)
ILW	intermediate-level radioactive waste (ILW)
HLW	high radioactive waste (HLW)
